# Protocol for the unclassified primary antibody deficiency (unPAD) study: Characterization and classification of patients using the ESID online Registry

**DOI:** 10.1371/journal.pone.0266083

**Published:** 2022-03-25

**Authors:** Lisanne M. A. Janssen, Ineke C. G. M. Reijnen, Cinzia Milito, David Edgar, Helen Chapel, Esther de Vries, the unPAD consortium

**Affiliations:** 1 Department of Tranzo, TSB, Tilburg University, Tilburg, the Netherlands; 2 Department of Pediatrics, Amalia Children’s hospital, Nijmegen, the Netherlands; 3 Department of Pediatrics, Elisabeth-Tweesteden Hospital, Tilburg, the Netherlands; 4 Department of Molecular Medicine, Sapienza University of Rome, Rome, Italy; 5 Department of Immunology, St James’s Hospital, Dublin & (ii) Trinity College, Dublun, Ireland; 6 Primary Immunodeficiency Unit, Nuffield Department of Medicine, University of Oxford, Oxford, United Kingdom; 7 Laboratory of Medical Microbiology and Immunology, Elisabeth-Tweesteden Hospital, Tilburg, the Netherlands; Sohag University Faculty of Medicine, EGYPT

## Abstract

**Background:**

Primary antibody deficiencies (PADs) without an identified monogenetic origin form the largest and most heterogeneous group of primary immunodeficiencies. These patients often remain undiagnosed for years and many present to medical attention in adulthood after several infections risking structural complications. Not much is known about their treatment, comorbidities, or prognosis, nor whether the various immunological forms (decreased total IgG, IgG subclass(es), IgM, IgA, specific antibody responses, alone or in combination(s)) should be considered as separate, clearly definable subgroups. The unclassified primary antibody deficiency (unPAD) study aims to describe in detail all PAD patients *without* an identified specific monogenetic defect regarding their demographical, clinical, and immunological characteristics at presentation and during follow-up. In constructing these patterns, the unPAD study aims to reduce the number of missed and unidentified PAD patients in the future. In addition, this study will focus on subclassifying unPAD to support the identification of patients at higher risk for infection or immune dysregulation related complications, enabling the development of personalized follow-up and treatment plans.

**Methods and analysis:**

We present a protocol for a multicenter observational cohort study using the ESID online Registry. Patients of all ages who have given informed consent for participation in the ESID online Registry and fulfill the ESID Clinical Working Definitions for ‘unclassified antibody deficiency’, ‘deficiency of specific IgG’, ‘IgA with IgG subclass deficiency’, ‘isolated IgG subclass deficiency’, ‘selective IgM deficiency’, ‘selective IgA deficiency’ or ‘common variable immunodeficiency’ will be included. For all patients, basic characteristics can be registered at first registration and yearly thereafter in level 1 forms. Detailed characteristics of the patients can be registered in level 2 forms. Consecutive follow-up forms can be added indefinitely. To ensure the quality of the collected data, all data will be fully monitored before they are exported from the ESID online Registry for analysis. Outcomes will be the clinical and immunological characteristics of unPAD at presentation and during follow-up. Subgroup analyses will be made based on demographical, clinical and immunological characteristics.

## Introduction

Ear-nose-throat (ENT) and lower airway symptoms occur commonly in the general population; they are often, but not always, caused by infection. These infections already start early in life, are mostly viral in origin and self-limiting. When symptoms continue to recur, allergy, asthma, smoking and/or (in adults) chronic obstructive pulmonary disease (COPD) can be the underlying cause [1]. Only a small number of patients suffer from too many, too frequent, unusual and/or severe infections caused by inborn errors of immunity (IEI). The majority of IEI patients suffer from predominantly antibody deficiencies (PAD), which are generally not immediately life-threatening. PADs can be subdivided into the rare, more severe, agammaglobulinemias and hyper-IgM syndromes, and the less rare hypogammaglobulinemias [2]. The latter may remain undiagnosed for years [2–5]; however, also these can ultimately lead to important morbidity, irreversible organ damage and reduced lifespan when they are not recognized and adequately treated in time [6–8].

Traditionally, common variable immunodeficiency disorders (CVID) are considered a separate PAD entity, comprising the most severe hypogammaglobulinemia patients [9,10]. CVID is the most common form seen in specialized centers (estimated prevalence in the population 1: 10.000–50.000) [11]. However, even for CVID, expert opinion varies as to which patients with decreased IgG and disturbed specific antibody responses should be classified under this diagnosis, some considering combination with decreased IgA *or* decreased IgM sufficient, and others diagnosing CVID *only* in case IgA is decreased (± decreased IgM) [12]. Many more patients suffer from less-well described and understood forms of hypogammaglobulinemia: decreased total IgG, IgG-subclass(es), IgM, IgA and/or specific antibodies, alone, or in combination(s) [2]. The International Union of Immunological Societies (IUIS) has grouped these cases together in the ‘predominantly antibody deficiencies’ section as ‘isotype/light chain/functional deficiencies’ (with a subdivision based on immunological laboratory values; Table 1) [3]; in the European Society for Immunodeficiencies (ESID) Clinical Working Definitions they are divided in separate entities which overlap in part with the IUIS subdivisions (Table 2) [13]. However, these PAD cases are often difficult to classify, either because aspects of more than one subgroup are found within the same patient, or because the patient’s immune capacity has not been sufficiently investigated to be positioned in a specific subgroup. They are therefore often referred to as “other hypogammaglobulinemia” or—more recently—as “unclassified primary antibody deficiency (unPAD)” [14]. Within this group, clinical severity as well as the results of immunological laboratory investigations and potential underlying pathophysiology may differ greatly. Also, different centers are inclined to treat the classification of these patients in different ways, making comparative studies difficult to perform.

**Table 1:** IUIS phenotypical classification–predominantly antibody deficiencies (without an identified monogenetic origin).

Phenotypical classification	Criteria
Hypogammaglobulinemia	
Common variable immunodeficiency (CVID) Phenotype (with no known disease-causing monogenic defect specified)	Decrease of IgG, IgA and/or IgM AND secondary causes of hypogammaglobulinemia have been excluded AND B cells > 1% Clinical phenotypes vary: most have recurrent infections, some have polyclonal lymphoproliferation, autoimmune cytopenias and/or granulomatous disease
**Other antibody deficiencies**	**Isotype, light chain or functional deficiencies with generally normal numbers of B cells**
IgG subclass deficiency with IgA deficiency	Recurrent bacterial infections May be asymptomatic Reduced IgA with decrease in one or more IgG subclass(es)
Isolated IgG subclass deficiency	Usually asymptomatic A minority may have poor antibody response to specific antigens and recurrent viral/bacterial infections Reduction in one or more IgG subclass(es)
Selective IgM deficiency	Pneumococcal/ bacterial infections Absent serum IgM
Selective IgA deficiency	May be asymptomatic Bacterial infections, autoimmunity mildly increased Very low to absent IgA with other isotypes normal, normal subclasses and specific antibodies
Specific antibody deficiency with normal immunoglobulin levels and normal B cells	Reduced ability to produce antibodies to specific antigens Immunoglobulin levels normal

Source: Bousfiha et al. [3].

**Table 2:** The Clinical Working Definitions for primary antibody deficiencies (without an identified monogenetic origin) in the ESID online registry.

**No.**	**Clinical Working Definition** ^**a**^	**Criteria**
1	**Common variable immunodeficiency disorders (CVID)**	Patients with at least one of the following: • Increased susceptibility to infection • Autoimmune manifestations • Unexplained granulomatous disease • Unexplained polyclonal lymphoproliferation • Affected family member with antibody deficiency AND marked decrease of IgG and marked decrease of IgA with or without low IgM levels (measured at least twice; <2SD of the normal levels for their age) AND at least one of the following: • Poor antibody response to vaccines (and/or absent Isohemagglutinins) • Low switched memory B cells (<70% of age-related normal value) AND secondary causes of hypogammaglobulinemia have been excluded AND diagnosis is established after the 4^th^ year of life AND no evidence of profound T-cell deficiency, defined as 2 out of the following (y = year of life): • CD4 numbers/microliter: 2-6y <300, 6-12y <250, >12y <200 • % naïve CD4: 2-6y <25%, 6-16y <20%, >16 <10% • T cell proliferation absent
2	**Deficiency of specific IgG (specific antibody deficiency–SPAD)**	Infections (recurrent or severe bacterial) AND normal serum/plasma IgG, A and M and IgG subclass levels AND profound alteration of the antibody responses to S. pneumonia (or other polysaccharide vaccine) either after documented invasive infection or after test immunization AND exclusion of T-cell defect
3	**IgA with IgG subclass deficiency**	Infections (recurrent or severe bacterial) AND undetectable serum/plasma IgA level (with normal/lowish IgG and IgM levels) AND low levels in one of more IgG subclass (documented twice) AND normal IgG antibody response to some vaccinations AND exclusion of T-cell defect
4	**Isolated IgG subclass deficiency**	Infections (recurrent or severe bacterial) AND normal IgG, A and M serum/plasma levels AND low levels in 1, 2, 3 IgG subclass or several missing (documented twice) AND normal IgG antibody response to some vaccinations AND exclusion of T-cell defect
5	**Selective IgM deficiency**	Infections (either invasive or recurrent, usually bacterial) AND low IgM serum/plasma level (with normal IgG and IgG subclasses and IgA plasma level) AND normal IgG antibody response to all vaccinations AND exclusion of T-cell defect
6	**Selective IgA deficiency**	At least one of the following: • Increased susceptibility to infection • Autoimmune manifestations • Affected family member AND diagnosis after 4^th^ year of life AND undetectable serum IgA, but normal serum IgG and IgM (measured at least twice) AND secondary causes of hypogammaglobulinemia have been excluded AND normal IgG antibody response to vaccination AND exclusion of T-cell defect
7	**Unclassified antibody deficiency** ^**b**^	Patients with at least 1 of the following 4: • Recurrent or severe bacterial infections • Autoimmune phenomena (especially cytopenia’s) • Unexplained polyclonal lymphoproliferation • Affected family member AND at least one of the following: • Marked decrease of at least one of total IgG, IgG1, IgG2, IgG3, IgA or IgM levels • Failure of IgG antibody response(s) to vaccines AND secondary causes of hypogammaglobulinemia have been excluded (infection, protein loss, medication, pregnancy) AND no clinical signs of T-cell related disease AND does not fit any of the other working definitions (excluding ‘unclassified immunodeficiencies’)

^a^ For this project, the combined patients under working definitions 2–7 are referred to as ‘unPAD patients’.

^b^ The criteria for working definitions 1–6 are very strict. All ‘predominantly antibody deficiencies’ that do not completely fulfil all criteria of any of these working definitions 1–6 should be registered under 7—unclassified antibody deficiency. If the patient does not completely fulfil all criteria for ‘unclassified antibody deficiency’ he/she should be registered under ‘unclassified immunodeficiency’ (if applicable; it is also possible that no immunodeficiency whatsoever is present).

Source: https://esid.org/Working-Parties/Registry-Working-Party/Diagnosis-criteria.

Because IEI are rare disorders, international collaboration is necessary to study these diseases. Since 2004, the ESID has been running an online database for primary immunodeficiencies: the ESID online Registry [15]. This database currently comprises information on more than 30,000 patients with errors of immunity. Documentation is organized in different levels. Level 1 is a basic dataset comprising the IEI diagnosis, demographic data, the way to diagnosis (including the presenting symptoms), immunoglobulin replacement therapy, hematopoietic stem cell transplantation and gene therapy. This level 1 information is meant for documentation of all patients who gave informed consent, with yearly concise follow-up documentation. An additional level 2 form was developed for more extensive long-term documentation of hypogammaglobulinemia patients which comprises a comprehensive dataset with additional items: additional clinical features, current and previous medications, diagnostic vaccinations, virological analyses, instrumental data (lung function, chest HRCT and gastroscopy), blood cell count, immunoglobulins, lymphocyte subsets, auto-antibodies, and further details on therapy.

Because of the moderately decreased immunoglobulin levels, unPADs are often considered to be clinically milder. However, unPAD-related symptoms can lead to decreased quality of life, loss of participation in society (school, work) and higher health care costs [6–8,16–18]. These people are often not recognized as IEI patients, because the general public as well as most health care professionals—who are not specialized in immunodeficiency—do not consider IEI in people with recurrent ‘normal’ infections. The concomitant fatigue these patients suffer is often considered to be of psychosocial origin or is interpreted as ‘chronic fatigue syndrome’.

We therefore initiated the unPAD study, based on the ESID online Registry, to describe in detail all types of PAD patients *without* an identified specific monogenetic origin (thus excluding e.g. X-linked and autosomal recessive agammaglobulinemia, and class-switch recombination defects) regarding their demographical, clinical and immunological characteristics at presentation and during follow-up, and to identify subgroups based upon the patterns in these characteristics which can support refining of the classification. By better characterization and classification of the disease, the unPAD study aims to support reducing the number of missed and unidentified PAD patients in the future. To ensure the quality of the collected data, all data will be fully monitored before they are exported from the ESID online Registry system for analysis. In this article, we describe in detail the design of the unPAD study, including the strict monitoring rules, and the planned statistical analysis of the obtained data.

## Methods

### Study objective

F*or this project* the current Clinical Working Definitions in the ESID online Registry ‘deficiency of specific IgG (specific antibody deficiency–SPAD)’, ‘IgA with IgG subclass deficiency’, ‘isolated IgG subclass deficiency’, ‘selective IgM deficiency’, ‘selective IgA deficiency’, and ‘unclassified primary antibody deficiency’ [13] will hereafter be referred to as ‘unPAD patients’. The unPAD study aims to characterize all types of PAD patients *without* an identified specific monogenetic origin, i.e. unPAD patients and patients fulfilling the Clinical Working Definition ‘common variable immunodeficiency (CVID)’ [14]. We will classify all included patients into subgroups with classification techniques using the demographical, clinical and/or immunological characteristics as directed by the best fit. Finally, we will analyze the predictive potential of demographical, clinical and/or immunological characteristics in relation to the occurrence of PAD-related complications such as bronchiectasis or cytopenias in both our newly defined hypogammaglobulinemia subgroups as well as in the subgroups based on the current Clinical Working Definitions.

### Study questions underlying the level 2 ESID Registry variables

A subset of the members of the ESID Registry Working Party formulated the research questions underlying the unPAD level 2 forms of the ESID online Registry in (mainly remote) consensus discussions:

1. What is the clinical presentation of these patients *at diagnosis* (spectrum, observed prevalence, subgroups, age-related differences)?
2. What is the immunological presentation of these patients *at diagnosis*?
3. Can subgroups be identified *at diagnosis* based on clinical and/or immunological characteristics?
4. What is the clinical presentation of these patients *during follow-up* (spectrum, observed prevalence, subgroups, age-related differences)?
5. What is the immunological presentation of these patients *during follow-up*?
6. Can subgroups be identified based on clinical and/or immunological characteristics; if so, is this a stationary classification, or do patients develop from one subgroup to another/others *with time*?

And in the long run:

7. What is the prognosis of (subgroups of) these patients regarding infections, complications, long-term sequelae, life expectancy, quality of life and ability to function in society?

### Patient eligibility

Before patient data can be entered into the ESID online Registry informed consent has to be obtained. The patient consent forms containing information on the ESID online Registry are available on the ESID website in many languages [19]. These forms need to be approved by a competent local Research Ethics Committee according to the regulations of the respective countries and documenting centers before use.

### Inclusion criteria

1. The patient (or parents in case of children) has given informed consent for participation in the ESID online Registry.
2. The patient fulfils the ESID online Registry Clinical Working Definitions for ‘unclassified antibody deficiency’, ‘deficiency of specific IgG (specific antibody deficiency–SPAD)’, ‘IgA with IgG subclass deficiency’, ‘isolated IgG subclass deficiency’, ‘selective IgM deficiency’, ‘selective IgA deficiency’ or ‘CVID’ (specified in Table 2).
3. At least the registration set of both level 1 and level 2 ‘at diagnosis’ forms has been completed.

### Exclusion criteria

1. Refusal of the reporting physician to have all data that were entered by the center in the ESID online Registry checked and–if necessary–corrected under supervision of the unPAD study monitor(s).
2. Patients with an identified monogenetic disease-causing mutation leading to reclassification.

### Study design

The unPAD study is an international multicenter observational cohort study based on the ESID online Registry data. Repeated calls for participation were published in the ESID Newsletter and on the ESID website. Furthermore, when participating centers indicated they knew of other centers who might be interested in participating, we contacted these centers. Until now, 20 centers from 10 countries actively participate in this study by collecting their data in the level 1 and level 2 forms of the ESID online Registry and have agreed to join the study (see list in the acknowledgments).

Analyses on variables at diagnosis will be conducted from 2022 onwards. The unPAD study is an ongoing study, there is still an open invitation for researchers in the field to participate in the study. The unPAD study will be running as long as the investigators expect additional information can be gained from another round of analysis, which will by nature mean a longer follow-up period than in the analyses performed before.

### Variables at baseline and during follow-up

For all patients, baseline characteristics are being registered at first registration and yearly thereafter in the so-called level 1 forms. The level 1 form contains data on demographic characteristics, family history, consanguinity, IEI diagnosis, and treatment (Table 3). More detailed characteristics of the patients can be registered in level 2 forms, including detailed data on demographical, clinical and immunological characteristics, including data on additional investigations, such as lung function, gastroscopy, and Chest CT-scan (Table 3). Consecutive follow-up forms can be added indefinitely (shown in S1 Table).

**Table 3:** Overview of variables included in the unPAD study.

Variable	Definition
General (level 1)	
**Patient**	
Patient consent	Signed/Not applicable (only if deceased) For minors, parents or the legal guardian must give their written consent.
Date of birth	Year; Month (month only if <12 years of age)
Country of current residence	This should be the country where the patient has his permanent residence, i.e. where he/she lives for the majority of the year. If the patient stays in the current country for a longer period, but only temporarily (e.g. for specialized medical treatment or seasonal work), his/her country of origin should be selected.
Sex	Male/Female
Familial case	Defined as another patient with a diagnosed primary immunodeficiency in the genetic family (e.g. parents, siblings, grandparents).
Consanguinity of parents	Defined as genetically related parents or other ancestors (e.g. grandparents) of the patient.
Documenting Centre	Name of the center from which the data originate.
**Way to Diagnosis**	
Date of first clinical diagnosis of IEI	Year; Month; Day The date when this patient was first diagnosed with a primary immunodeficiency based on clinical features and laboratory values.
First IEI-related symptom(s)	• Infection • Immune dysregulation (lymphoproliferation, granuloma formation, autoimmunity, inflammatory bowel disease, celiac disease, vasculitis, eczema, autoinflammatory disease) • Malignancy • Syndrome manifestations • Other • No IEI-related symptoms at all
Date of onset of symptoms	Year; Month The year and month when the first symptoms suggestive of an IEI (see above) appeared in this patient, based on the physician’s judgement.
**IEI Diagnosis**	
Current IEI Diagnosis	Defined as the most recent IEI diagnosis.
Affected gene	The gene in which disease-causing mutation(s) have been found in this patient.
**Status**	
Current status	• Alive • Deceased • Lost to follow-up • Discharged after complete recovery
Current Ig replacement	Yes/No
Did the patient ever receive immune modifying treatment?	Yes/No
Did the patient ever suffer from a malignancy?	Yes/No
HSCT	Yes/No
Splenectomy	Yes/No
Gene therapy	Yes/No
**unPAD study (level 2)**^a^ first registration	
Clinical presentations (multiple answer)	• Recurrent ENT and airway infections • Failure to thrive from early infancy • Recurrent pyogenic infections • Unusual infections or unusually severe course of infections • Recurrent infections with the same type of pathogen • Autoimmune or chronic inflammatory disease; lymphoproliferation
Clinically most important clinical presentation (single answer)	• Recurrent ENT and airway infections • Failure to thrive from early infancy • Recurrent pyogenic infections • Unusual infections or unusually severe course of infections • Recurrent infections with the same type of pathogen • Autoimmune or chronic inflammatory disease; lymphoproliferation
Bacterial infections	Any major bacterial infection (+ which micro-organism)? • Pneumonia • Meningitis • Osteomyelitis • Liver Abscess • Other major infection
Frequently recurring infections	• Upper respiratory tract • Lower respiratory tract • Gastrointestinal tract • Urinary tract • Skin • Other
Unusual infections	• Severe viral • Opportunistic • Parasitic
Inflammatory bowel disease/ allergic manifestations	Inflammatory bowel disease is subdivided in ‘biopsy-proven’ and ‘clinically suggestive, but not biopsy-proven’. Allergic manifestations are subdivided in ‘proven with sensitization’ and ‘clinically suggestive, but not proven by sensitization’.
Chronic organ pathology	• Hepatomegaly • Splenomegaly (splenectomy ever performed?) • Chronic liver disease • Bronchiectasis • Parenchymal lung disease • Hearing impairment (not congenital) • Other
Autoimmunity	• Auto-immune hemolytic anemia • Auto-immune granulocytopenia • Auto-immune thrombocytopenia • Other
Malignancy and other manifestations	The type of malignancy and/or of other manifestations has to be specifically defined.
Medication	Daily immunosuppressive drugs or drugs that may cause hypogammaglobulinemia as a side effect (currently in use or stopped less than three months before the diagnosis of hypogammaglobulinemia).
Diagnostic vaccination response measurements	• Tetanus • Pneumococcal polysaccharide • Other
Virological analysis	• HCV-RNA • HIV-DNA • EBV-DNA • CMV-DNA
Instrumental data	• Lung function; FEV1 • HRCT thorax • Gastroscopy
Blood counts/ Immunoglobulins/ sensitization	• Laboratory values at time point closest to the diagnosis (leukocytes, neutrophils, lymphocytes, eosinophils, basophils, monocytes) • Laboratory values at time point closest to diagnosis before start of Ig-replacement (IgG, IgG1, IgG2, IgG3, IgG4, IgA, IgM, IgE, M-protein) • Sensitization (specific IgE, skin prick test)
Lymphocyte subsets/ auto-anti-bodies	• Laboratory values at time point closest to diagnosis (CD3+, CD3+CD4+, CD3+CD8+, CD19+CD20+, CD3-CD16/56+, CD20+CD27+IgD-, CD19+CD38++IgM++, CD19+CD27-IgM+IgD+, CD19+CD27+IgM+IgD+, CD19+CD27+IgM+IgD-, CD19+CD27+IgM-IgD-) • Auto-antibodies (ANA, TPO-antibodies)

^a^ Follow-up forms (shown in S1 Table) can be added indefinitely.

Abbreviations: ANA, antinuclear antibody; CD, cluster of differentiation; CMV, cytomegalovirus; DNA, deoxyribonucleic acid; EBV, Epstein-Barr Virus; e.g., exempli gratia; ENT, ear-nose-throat; IEI, inborn error of immunity; FEV1, forced expiratory volume in 1 second; HCV, hepatitis C virus; HIV, human immunodeficiency virus; HRCT, high-resolution computed tomography; HSCT, hematopoietic stem cell transplantation; Ig, immunoglobulin; RNA, ribonucleic acid; TPO, thyroid peroxidase; unPAD, unclassified primary antibody deficiency.

### Data collection and storage

The registered patient data are stored on secure servers at the University Hospital Freiburg, Freiburg, Germany, using a study code. Data transfer is SSL encrypted. These pseudonymized data can only be traced back to the patient by the treating physician or documentation specialist of the center in question, not by the unPAD research team, following the European legal data protection provisions. Identifying data (e.g., name, place of residence) are stored on a separate server to which third parties have no access. The system structure of the ESID online database has been described by Perner et al. and Guzman et al [15,20]. Before registration of patient data is possible, a participating center must have signed a contract and obtained logins for the database system. The database is designed to be used for long-term documentation. It offers the possibility to add any number of visit dates for a given patient. Participating centers are asked to update their patients’ data at least once a year. The database has an inbuilt automatic quality assurance system including field type, range and plausibility checks (e.g., date of death must be later than date of birth). Some fields are mandatory, which means that data cannot be stored unless these fields are completed. Taking into account that the data are sometimes not known or currently not available to the documentalist, the boxes ‘truly unknown’ or ‘currently unknown’ can be checked. All patient data collected in the level 1 and level 2 forms will be fully monitored before data extraction for analysis in the unPAD study. In case of missing data or inconsistencies, the unPAD research team will contact the participating centers to resolve these issues.

### Sample size

In order to be able to accurately describe unPAD patients, we aim to collect data on as many patients as possible. Based on the amount of registered unPAD patients in the ESID online Registry, we aim to include at least 1,000 patients. This number will allow analysis of the demographical, clinical, and immunological characteristics (at presentation and during follow-up) and of the risk of complications in potentially meaningful subgroups.

### Statistical analysis

Statistical analyses will be performed with IBM SPSS Statistics and/or R (most recent versions). Data quality will be secured by the thorough monitoring process before data extraction. After extraction, the data will be cleaned and preprocessed supported by the standard set of descriptive statistics plus visualization techniques. The most suitable method for dealing with missing variables will be determined for each variable in collaboration between data analysts and domain experts (e.g., types of imputation, exclusion from analyses). We will use cluster analysis (with bootstrapping) plus supervised and unsupervised machine learning for subgroup classification using all variables together as well as (combinations of) subsets of demographical, clinical and immunological characteristics. In addition, we will use regression analysis and machine learning to create and evaluate models for predicting health-related outcome variables such as bronchiectasis. Appropriate evaluation metrics will be applied for these models depending on their type, such as R^2^, accuracy, mean absolute error (MAE), (root) mean squared error ((R)MSE), and area under the receiver operating characteristic curve (ROC-AUC). A p-value <0.05 with correction for multiple testing when appropriate will be considered statistically significant, and/or a 95% confidence interval (CI) not containing 0, where applicable.

## Discussion

Most hypogammaglobulinemia patients, including those with CVID, still lack a definitive genetic diagnosis. The unPAD study has been designed to investigate ‘unclassified antibody deficiency’ and has the intention to describe in detail all types of PAD patients *without* an identified specific monogenetic disease-causing mutation regarding their demographical, clinical, and immunological characteristics at presentation and during follow-up. UnPAD patients form a highly heterogenous group and will remain so unless classification into clinically meaningful subgroups can be made. Efforts to stratify patients into different subgroups according to genetic screening, B- and T-cell studies [21–25] and clinical presentations [26] have been made for CVID patients. A larger group of patients suffers from a range of combinations of immunoglobulin deficiencies where the CVID definition is not met (referred to in the literature as idiopathic hypogammaglobulinemia [27], CVID-like disorder [28], IgG isotype deficiency [29], or unclassified hypogammaglobulinemia [30], and by us as unPAD). However, efforts to stratify patients into different subgroups have not yet been made for these patients. Because these disorders form a heterogenous and phenotypically overlapping group, correct classification is a real challenge. It is important to realize that current classifications (ESID Clinical Working Definitions, IUIS) are mainly based upon the results of immunological laboratory investigations, while it is not clear how *clinically* useful such a basis for classification really is. In addition to the current laboratory classification approach, we therefore plan a new, broader clinical classification approach. By grouping patients also based on clinical presentations and complications we aim to subclassify unPAD patients to support identification of those patients with higher risks of complications. These patients could then be monitored for specific complications or be treated differently according to subtype. This will ultimately shed light on more personalized intervention approaches. In addition, the potential identification of more homogenous subgroups can help to unravel the genetic background of unPAD patients. This information will help to guide clinicians to answer the question: “*what should I do with this individual unPAD patient*?”.

This is important. Although doctors are inclined to consider patients with hypogammaglobulinemia who do not match the CVID diagnostic criteria to be clinically mild, CVID and unPAD patients comprise phenotypically overlapping groups. On the one hand, the often milder affected ‘infection-only’ group of CVID patients share very similar disease courses to patients currently classified as unPAD. On the other hand, certain subgroups of unPAD patients suffer from similar immune dysregulation features as described in CVID [14]. The unPAD study can improve PAD patient care by identifying subgroups at risk for serious complications, implying different therapeutic consequences for these patients.

The unPAD study will be the largest study on unPAD patients to date. Of all centers participating in the ESID online Registry, 20 have indicated to participate in the unPAD study so far (13 pediatric and 7 adult centers). Of these, 10 centers have already been fully monitored during a site visit, resulting in 1010 patients who have been monitored at this moment. This was done as preliminary work to find out whether we would achieve sufficient statistical power. This large set of patient data provides significant statistical power to not only describe the clinical presentation, prognosis, and treatment of unPAD in detail, but also to determine whether subgroups can be identified based on demographical, clinical, and immunological characteristics.

The unPAD study has its limitations. Due to lack of international consensus, the local diagnostical, treatment and follow-up protocols may differ between centers. For instance, not all patients will have undergone complete pulmonary examinations (e.g., spirometry and chest HRCT), which may lead to an underestimation of the frequency of bronchiectasis or interstitial lung disease. There will be variability in data entry practices: e.g. some centers will only record IgA deficiency if patients require active management and the adherence with annual data updating will be dependent on available resources. Moreover, facilities for genetic testing differ between centers. Therefore, a subgroup of patients with a *non*-identified genetic diagnosis may be hidden in the clinically defined unPAD cohort who should actually be reclassified to a monogenetic IEI form.

The most important strength of the study is that *all* data will be monitored and–if necessary–corrected and supplemented. The usefulness and quality of data extracted from patient registries depends on correct data entry. It is thus of utmost importance for the data quality assurance to review and check the data of any newly added patient. Problems that can occur during registration of PAD patient data are, for example, entering incorrect numbers of immunoglobulins and lymphocyte subpopulations by typing errors, using wrong units (cells/ul instead of 10^9^/l in lymphocyte subpopulations), misinterpretation of vaccine responses and incomplete clinical manifestations hidden under ‘other options’. Furthermore, the ESID online Registry can only indicate whether a gastroscopy or chest HRCT-scan has been performed, and if so, whether the result was normal or abnormal, but the exact findings cannot be registered in the system. A monitor site visit provides the opportunity to also retrieve these detailed data, which can provide very valuable additional information.

The unPAD study is an ongoing study and explicitly reaches out to other researchers and clinicians in the field of PAD to join the study. This initiative aims to become a platform that facilitates future collaborative research in the field. We expect that our study will give more insight in the demographical, clinical, and immunological characteristics of unPAD patients and will identify which subgroups are at risk for infections or complications based on immune dysregulation, enabling the development of personalized follow-up and treatment plans.

## Supporting information

S1 Table Overview of variables included in the follow-up forms of the ESID online Registry used in the unPAD study. ^a^ Follow-up forms can be added indefinitely. Abbreviations: ANA, antinuclear antibody; CD, cluster of differentiation; CMV, cytomegalovirus; DNA, deoxyribonucleic acid; EBV, Epstein-Barr Virus; e.g., exempli gratia; ENT, ear-nose-throat; IEI, inborn error of immunity; FEV1, forced expiratory volume in 1 second; HCV, hepatitis C virus; HIV, human immunodeficiency virus; HRCT, high-resolution computed tomography; HSCT, hematopoietic stem cell transplantation; Ig, immunoglobulin; RNA, ribonucleic acid; TPO, thyroid peroxidase; unPAD, unclassified primary antibody deficiency.
(DOCX)
