# Supplementary material for: Protocol for the unclassified primary antibody deficiency (unPAD) study: Characterization and classification of patients using the ESID online Registry
Source: PLoS One. 2022 Mar 25;17(3):e0266083. doi: 10.1371/journal.pone.0266083 (PMC9045688; doi:10.1371/journal.pone.0266083)
Supplement: S1 Table — a Follow-up forms can be added indefinitely. Abbreviations: ANA, antinuclear antibody; CD, cluster of differentiation; CMV, cytomegalovirus; DNA, deoxyribonucleic acid; EBV, Epstein-Barr Virus; e.g., exempli gratia; ENT, ear-nose-throat; IEI, inborn error of immunity; FEV1, forced expiratory volume in 1 second; HCV, hepatitis C virus; HIV, human immunodeficiency virus; HRCT, high-resolution computed tomography; HSCT, hematopoietic stem cell transplantation; Ig, immunoglobulin; RNA, ribonucleic acid; TPO, thyroid peroxidase; unPAD, unclassified primary antibody deficiency. (DOCX) [file pone.0266083.s001.docx]

**Supplementary Table 1. Overview of variables included in the follow-up forms of the unPAD study**

| **Variable** (since last documentation) | **Definition** |
| --- | --- |
| **unPAD study (level 2)**^a^ |  |
| Follow-up | - Visit date - Current weight and Height, BMI |
| Bacterial infections | Any major bacterial infection (+ which micro-organism)?   - Pneumonia - Meningitis - Osteomyelitis - Liver Abscess - Other major infection |
| Frequently recurring infections^b^ | - Upper respiratory tract - Lower respiratory tract - Gastrointestinal tract - Urinary tract - Skin - Other |
| Unusual infections | - Severe viral - Opportunistic - Parasitic |
| Inflammatory bowel disease/ allergic manifestations | Inflammatory bowel disease is subdivided in ‘biopsy-proven’ and ‘clinically suggestive, but not biopsy-proven’. Allergic manifestations are subdivided in ‘proven with sensitization’ and ‘clinically suggestive, but not proven by sensitization’. |
| Chronic organ pathology | - Hepatomegaly - Splenomegaly (splenectomy ever performed?) - Chronic liver disease - Bronchiectasis - Parenchymal lung disease - Hearing impairment (not congenital) - Other |
| Autoimmunity | - Auto-immune haemolytic anaemia - Auto-immune granulocytopenia - Auto-immune thrombocytopenia - Other |
| Malignancy and other manifestations | The type of malignancy and/or of other manifestations has to be specifically defined. |
| Medication | Daily immunosuppressive drugs or drugs that may cause hypogammaglobulinemia as a side effect (currently in use or stopped less than three months before this documentation). |
| Diagnostic vaccinations | - Tetanus - Pneumococcal polysaccharide - Other |
| Virological analysis | - HCV-RNA - HIV-DNA - EBV-DNA - CMV-DNA |
| Instrumental data | - Lung function; FEV1 - HRCT thorax - Gastroscopy |
| Blood counts/ Immunoglobulins/ sensitization | - Laboratory values at time point closest to this documentation (leukocytes, neutrophils, lymphocytes, eosinophils, basophils, monocytes) - Laboratory values at time point closest to this documentation (IgG, IgG1, IgG2, IgG3, IgG4, IgA, IgM, IgE, M-protein) - IgG measured under Ig substitution (Yes/No/Unknown) - Sensitization (specific IgE, skin prick test) |
| Lymphocyte subsets/ auto-anti-bodies | - Laboratory values at time point closest this documentation (CD3+, CD3+CD4+, CD3+CD8+, CD19+CD20+, CD3-CD16/56+, CD20+CD27+IgD-, CD19+CD38++IgM++, CD19+CD27-IgM+IgD+, CD19+CD27+IgM+IgD+, CD19+CD27+IgM+IgD-, CD19+CD27+IgM-IgD-) - New results auto-antibodies (ANA, TPO-antibodies) |

*^a^ Follow-up forms can be added indefinitely.*

*^b^Defined as acute respiratory infections occurring 8 episodes per year if age < 3 years and/or 6 episodes per year if age ≥ 3 years.*

*Abbreviations: ANA, antinuclear antibody; CD, cluster of differentiation; CMV, cytomegalovirus; DNA, deoxyribonucleic acid; EBV, Epstein-Barr Virus; e.g., exempli gratia; ENT, ear-nose-throat; IEI, inborn error of immunity; FEV1, forced expiratory volume in 1 second; HCV, hepatitis C virus; HIV, human immunodeficiency virus; HRCT, high-resolution computed tomography; HSCT, hematopoietic stem cell transplantation; Ig, immunoglobulin; RNA, ribonucleic acid; TPO, thyroid peroxidase; unPAD, unclassified primary antibody deficiency.*
